# Dynaval: new product launch

**Published:** 2018

Pharma Dynamics, South Africa’s leading supplier of cardiovascular medicines, announces the addition of Dynaval (valsartan) to its extensive range ofcardiovascular medicines. Dynaval is indicated:

- for the treatment of mild to moderate hypertension
- to improve survival following myocardial infarction
- for the treatment of heart failure (NYHA class II–IV).

Dynaval is available in 80- and 160-mg tablets and is the only valsartan brand conveniently packed in 30 tablets.

**Table:** 

**Product**	**Active**	**Pack**	**Price (SEP excl VAT)**
Dynaval 80 mg	valsartan 80 mg	30 tabs	R92.66
Dynaval 160 mg	valsartan 160 mg	30 tabs	R92.66

For further information, kindly contact Afzal Dhansay, GroupProduct Manager: Cardiovascular at (021) 707-7000.

